# Hemangiopericytoma of the tongue

**DOI:** 10.1590/S1808-86942012000200024

**Published:** 2015-10-20

**Authors:** Carlos Eduardo Molinari Nardi, Victor Bandini Vieira, Elio Gilberto Pfuetzenreiter, Rogério Aparecido Dedivitis

**Affiliations:** a Head and Neck Surgery Resident – Hospital Ana Costa, Santos/SP, Brazil; b Medical Student – Universidade Metropolitana de Santos; c MSc in Health Sciences – Graduate Program of the Hospital Heliópolis HOSPHEL, São Paulo/SP. Professor of Surgery – Fundação Lusíada UNILUS, Santos/SP. Assistant- Head and Neck Surgery Services – Hospital Ana Costa and at the Irmandade da Santa Casa da Misericórdia de Santos; d MD. Senior Associate Professor – Fundação Lusíada UNILUS. Serviço de Cirurgia de Cabeça e Pesoço do Hospital Ana Costa, Santos/SP, Brasil

**Keywords:** hemangiopericytoma, immunohistochemistry, tongue neoplasms

## INTRODUCTION

Hemangiopericytoma is a rare type of tumor, which was first described in 1942 by Stout & Murray^1^, ^2^. It is believed that the hemangiopericytoma stems from vascular cells called Zimmerman pericytes. These pericytes are found throughout the entire spiral body which involves the capillars and post-capillary venules^3^. There is a predilection for the muscle-skeletal system^4^. It represents about 1% of all the vascular tumors^2^, and it usually affects adults^4^. Clinically, it affects any age, having a greater incidence between the third and sixth decades of life, without any gender predilection. It usually courses with slow and painless growth^2^. We describe here the case of 34-year-old patient with this tumor in the oral cavity.

## CASE REPORT

**Figure 1:**
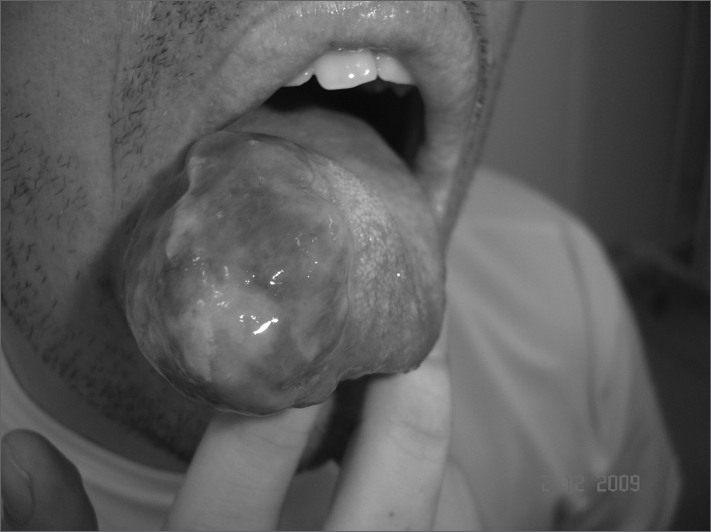
Tumoral mass in the right-side tongue border.

A 34-year old male patient with a lesion on the right tongue border, with two months of onset, with slow and progressive growth (Figure 1). He had been previously treated with cephalexin in another clinic, for seven days, without improvement. We chose the excisional biopsy and completely resected the lesion, which was well outlined upon surgery. The microscopic exam showed an amorphous mass, of gelatinous consistence, white-opaque color, with dark brown areas. The microscopic exam showed an ulcerated nodular structure made up of spindle-like cells arranged in bundles with uniform nuclei and low mitotic activity. There were areas with blood vessel proliferation. Immunohistochemistry showed a positive reaction towards the following markers: CD34, actin and factor VIII, yielding the diagnosis of hemangiopericytoma. The macro and microscopic margins were free, reason why no adjuvant therapy was required. The patient did not show evidence of disease after 18 months of follow up.

## DISCUSSION

The hemangiopericytoma is uncommon in the head and neck^2^. Stout & Murray (1942) described 691 cases of vascular tumors, and only nine of them were hemangiopericytomas^1^. Since then, there are approximately 300 cases of hemangiopericytomas described, especially on the trunk and lower limbs^2^. Only 15% to 30% of these tumors are found in the head and neck^3^. At this location, it affects mainly the soft tissue surrounding the oral cavity, sinusal tract and meninges and, more rarely, the orbit, parotid gland, skull base and temporal bone^2^.

Angiographic characteristics may help differentiate hemangiopericytomas from other types of hypervascularized tumors. Image studies, such as radiographies, CT scans and angiography are not specific. MRI reveals a solid mass with isodense contrast in T1^2^. Enzinger reported the following characteristics which match a high grade tumor: nuclear atypia, necrosis, hemangioma, four mitosis per microscopic field, and size greater than 6.5 cm^2^.

The differential diagnosis of highly vascularized tumors in the head and neck is a challenge, especially because of the difficulty in differentiating hemangiopericytomas from other tumors which have a prominent vascularization^2^. The differentiation of the hemangiopericytoma with the solitary fibrous tissue is complicated because of its marked morphology and similar immunohistochemistry. Positiveness for antigens CD-99 and bCl-2 is similar to that of solitary fibrous tumor; nonetheless, CD-34 varies its reaction and is not inconstantly positive for hemangiopericytoma^5^.

The treatment of choice is complete surgical resection of the lesion. Adjuvant radiotherapy and chemotherapy may be indicated in cases in which there is only a partial resection^2^.

Recurrences and distant metastases are rare in patients treated with complete surgical excision; nonetheless, most of the patients who had metastases or recurrences were diagnosed after over 40 months of follow up; suggesting a long standing postoperative follow-up for all the patients^3^.
